# Lgr5 Marks Post-Mitotic, Lineage Restricted Cerebellar Granule Neurons during Postnatal Development

**DOI:** 10.1371/journal.pone.0114433

**Published:** 2014-12-10

**Authors:** Tyler E. Miller, Jun Wang, Kumar Sukhdeo, Craig Horbinski, Paul J. Tesar, Robert J. Wechsler-Reya, Jeremy N. Rich

**Affiliations:** 1 Department of Stem Cell Biology and Regenerative Medicine, Lerner Research Institute, Cleveland Clinic, Cleveland, Ohio, United States of America; 2 Department of Pathology, Case Western Reserve University School of Medicine, Cleveland, Ohio, United States of America; 3 Sanford-Burnham Medical Research Institute, La Jolla, California, United States of America; 4 Department of Pathology and Laboratory Medicine, University of Kentucky, Lexington, Kentucky, United States of America; 5 Department of Genetics and Genome Sciences, Case Western Reserve University School of Medicine, Cleveland, Ohio, United States of America; 6 Department of Molecular Medicine, Cleveland Clinic Lerner College of Medicine of Case Western Reserve University, Cleveland, Ohio, United States of America; University of Kentucky, United States of America

## Abstract

Wnt signaling regulates self-renewal and fate commitment of stem and progenitor cells in development and homeostasis. Leucine-rich repeat-containing G-protein coupled receptor 5 (Lgr5) is a co-receptor for Wnt signaling that marks highly proliferative stem and progenitor cells in many epithelial tissue types. Wnt signaling instructs neural developmental and homeostatic processes; however, Lgr5 expression in the developing and adult brain has not been characterized. Here we report that Lgr5 is expressed in the postnatal cerebellum during the maturation and synaptogenesis of cerebellar granule neurons (CGNs), processes controlled by Wnt signaling. Using a transgenic reporter mouse for *in vivo* Lgr5 expression analysis and lineage tracing, we reveal that Lgr5 specifically identified CGNs and was restricted temporally to the CGN maturation phase within the internal granule layer, but absent in the adult brain. Cells marked by Lgr5 were lineage restricted, post-mitotic and long-lived. The ligand for Lgr5, R-spondin, was secreted in a paracrine fashion that evolved during the maturation of CGNs, which coincided with the Lgr5 expression pattern. Our findings provide potential new insight into the critical regulation of Wnt signaling in the developing cerebellum and support a novel role for Lgr5 in the regulation of post-mitotic cells.

## Introduction

The discovery of Lgr5 (leucine-rich-repeat-containing G-protein-coupled receptor 5) as an adult epithelial stem cell marker led to interest in this previously orphan receptor. Since its discovery as a marker of intestinal crypt stem cells [1], Lgr5 has been shown to mark stem or progenitor cell populations across diverse epithelial tissues, including skin, stomach, intestine, mammary gland, and cochlear hair follicle, and acts as a Wnt co-receptor (reviewed in [2]). To date, characterized epithelial Lgr5-positive cell populations all demonstrate self-renewal and proliferative capacity.

Wnt signaling plays an important role in many processes during development and homeostasis in the brain [3], [4]. However, Lgr5 expression and function as an important Wnt co-receptor in Wnt-dependent cell types in the brain has not been described. Cerebellar granule neurons (CGNs) make up the largest neuronal population in the mammalian brain, outnumbering all other neurons combined [5], and their development is dependent on Wnt signaling, suggesting that Lgr5 may contribute to CGN biology.

CGNs proceed through well-organized, sequential differentiation events during development [6]. During murine embryonic development, CGN precursors (CGNPs) from the rhombic lip migrate to form the external germinal layer (EGL), where they undergo extensive proliferation in response to Sonic hedgehog (Shh) secreted from Purkinje neurons. CGNP proliferation continues for the first two postnatal weeks, but within a few days of birth cells begin to exit the cell cycle and differentiate. CGNPs stop dividing to differentiate and migrate through the molecular layer into the internal granule layer (IGL) [7]. The final maturation phase occurs in the IGL when CGNs form branched dendrites and long axons – a Wnt signaling dependent process [8], [9]. CGNs secrete Wnt-7a, which acts in an autocrine fashion through the Frizzled-5 receptor to mediate synapse formation with excitatory mossy fibers [9], [10]. Proper development of CGNs is critically important to the overall development and architecture of the cerebellum. Abnormal development or loss of CGNs leads to severe cerebellar abnormalities in mice and several disease states in humans [11], [12]. Aberrant Wnt signaling in CGN precursors leads to severe cerebellar alterations [13], while interruption of Wnt signaling leads to improper synapse formation [9].

In other cell types, the Wnt receptor complex, consisting of LRP and Frizzled proteins, is recruited by and bound to R-spondin-activated Lgr5. Once bound to Lgr5, the LRP-Frizzled complex binds Wnt ligands to increase signaling through the Wnt/β-catenin pathway [14], [15]. However, the role of Lgr5 in CGN development is unknown.

Here we report that Lgr5 is expressed in CGNs exclusively during their Wnt-dependent maturation phase, and that the Lgr5 ligand, R-spondin1 (Rspo1) displays a spatio-temporal concomitant pattern of expression. These data indicate Lgr5 is involved in the orchestrated development of these non-stem neuronal cell populations, demonstrating a potential role for Lgr5 outside of epithelial stem cells.

## Materials And Methods

### Animals

This study was carried out in strict accordance with the recommendations in the Guide for the Care and Use of Laboratory Animals of the National Institutes of Health. All animal experiments were approved by the Cleveland Clinic Institutional Animal Care and Use Committee or the Sanford-Burnham Medical Research Institute Institutional Animal Care and Use Committee. *Lgr5-eGFP-IRES-CreER2* (*Lgr5^EGFP-CreERT2^*)(RRID:IMSR_JAX:008875)[1] and *Rosa26-lox-STOP-lox-tdTomato (R26R^tdTomato^*)(RRID:IMSR_JAX:007914)[16] mice were acquired from Jackson Labs. Standard PCR genotyping was performed according to the vendor's website. At least 3 *Lgr5^EGFP-CreERT2^* mice were examined for each time point and marker. *Catnb^flox(exon3)^* mice (RRID:MGI_MGI:2673882) [17] were previously described [18].

### Lgr5 lineage tracing

Mice heterozygous for *Lgr5^EGFP-CreERT2^* were crossed with the *R26R^tdTomato^* inducible reporter strain to breed *Lgr5^EGFP-CreERT2^; R26R^tdTomato^* offspring. Lineage tracing was achieved by intraperitoneal (i.p.) administration of tamoxifen to *Lgr5^EGFP-CreERT2^; R26R^tdTomato^* mice at 133 mg/kg body weight at P4 and P7 and 200 mg/kg at other indicated developmental time points [19]. At least 3 *Lgr5^EGFP-CreERT2^; R26R^tdTomato^* were examined for each time point and indicated marker.

### Constitutive Wnt-Activation in Lgr5-positive cells

Heterozygous *Lgr5^EGFP-CreERT2^* animals were crossed with *Catnb^flox(exon3)^* mice [17] to yield *Lgr5^EGFP-CreERT2^*; *Catnb^flox(exon3)^* (RRID:MGI_MGI:5475209) mice. To initiate constitutive activation of Wnt signaling, P4 pups were administered with 0.6 mg per 30 µL per pup tamoxifen through oral gavage. Mice were sacrificed by cardiac perfusion and whole brains dissected at P25 when the mice are afflicted with skin and intestinal abnormalities.

### Tissue processing, histology and imaging

Tissues were fixed in 4% PBS-buffered paraformaldehyde overnight and then transferred to 30% sucrose (w/v) for another 24 hours. Tissue was then embedded in Optimal Cutting Temperature (OCT) compound and sectioned at 8 microns on a Leica cryostat. Sections were blocked (PBS, 5% goat serum, 0.1% TritonX-100), incubated with primary antibodies for 2 hrs at room temperature in humidity chamber, washed with PBS and incubated with secondary antibodies for 45 min room temperature. Primary antibodies used: GFP (1∶250; Aves Labs Cat# GFP-1020 RRID:AB_10000240), Gabra6 (1∶100; EMD Millipore Cat# AB5610 RRID:AB_91935), NeuN Clone A60 (1∶100; Millipore Cat# MAB377 RRID:AB_2298772), BLBP (1∶250; EMD Millipore Cat# ABN14 RRID:AB_10000325), Calb1 D28K (1∶100; Sigma-Aldrich Cat# C9848 RRID:AB_476894), Olig2 (1∶150; EMD Millipore Cat# AB9610 RRID:AB_570666), Ki67 (1∶100; Abcam Cat# ab15580 RRID:AB_443209), Rspo1 (1∶1000; Sigma-Aldrich Cat# SAB3500046 RRID:AB_10602510), Pax6 (1∶250; Covance Cat# PRB-278P), Prominin-1 (1∶100; eBioscience Cat# 14-1331-90), Nestin (1∶300; BD Biosciences Cat#556309). Species-specific Alexa-Fluor-conjugated secondary antibodies were used for detection (1∶250; Invitrogen). tdTomato expression was visualized directly.

### Image Analysis

Image quantification was done in conjunction with ImageIQ (http://www.image-iq.com/) using image analysis software. Briefly, for Lgr5-GFP/tdTomato overlap experiments, Lgr5-GFP and tdTomato thresholds were set as standard across images and individual cells were marked separately as either negative or positive for each marker and then overlaid to quantify single- and double-positive cells.

## Results

### Lgr5 is expressed in the postnatal cerebellum and its expression is temporally restricted

**Figure 1:**
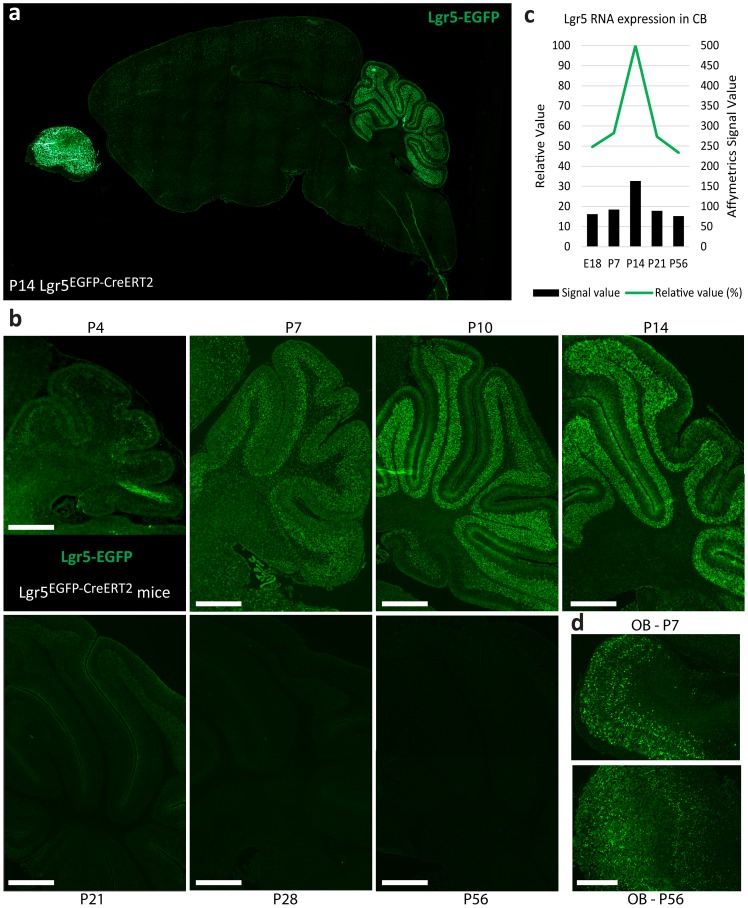
Lgr5 is expressed in the cerebellum during postnatal development. Sagittal brain immunofluorescence sections from *Lgr5^EGFP-CreERT2^* mice stained for EGFP to mark Lgr5+ cells at multiple time points. **a**) Tiled 10x magnified images of a midline sagittal section were stitched together to reveal Lgr5-EGFP expression at the P14 time point. **b**) Lgr5-EGFP expression at P4, P7, P10, P14, P21, P28 and P56 in the cerebellum demonstrating that Lgr5 expression turns on at P4, ramps up expression until its peak from P10-P14 and then expression is lost permanently over the next 7–14 days. **c**) Microarray expression data of Lgr5 generated from the Cerebellar Development Transcriptome Database [20] taken from total cerebellum RNA at indicated time points. Data acquired from http://www.cdtdb.neuroinf.jp/CDT/ReferTemporal.do?cdIdCh=CD12762.1. **d**) Olfactory Bulb (OB) at P7 and P56 reveal Lgr5-EGFP expression throughout development and adulthood. Scale bars, 400 microns.

We analyzed brain expression of Lgr5, a known marker of epithelial stem cells [2], to understand if Lgr5 was playing a role in neural stem and progenitor cells. We utilized the *Lgr5^EGFP-CreERT2^* mouse, in which EGFP expression is driven by the Lgr5 locus [1]. While we did not find significant expression of Lgr5 in the neural stem cell compartments (S1 Figure), we surprisingly found robust expression in the cerebellum and olfactory bulb in the developing postnatal brain (Fig. 1a). Cerebellar Lgr5 expression was temporally restricted to the period of postnatal development between postnatal day (P) 4 and P21, with maximum expression between P10 – P14 (Fig. 1b). This expression pattern was confirmed using RNA expression data from the Cerebellar Development Transcriptome Database (RRID:nif-0000–00008) [20], suggesting this phenomenon is not an artifact of the transgene (Fig. 1c). In contrast, Lgr5 is expressed in the olfactory bulb by postnatal day 4 and persists into adulthood (Fig. 1d). Overall, these results suggest specific and tight regulatory control of Lgr5 during development of the cerebellum.

### Lgr5-positive cells in the postnatal cerebellum are exclusively cerebellar granule neurons

**Figure 2:**
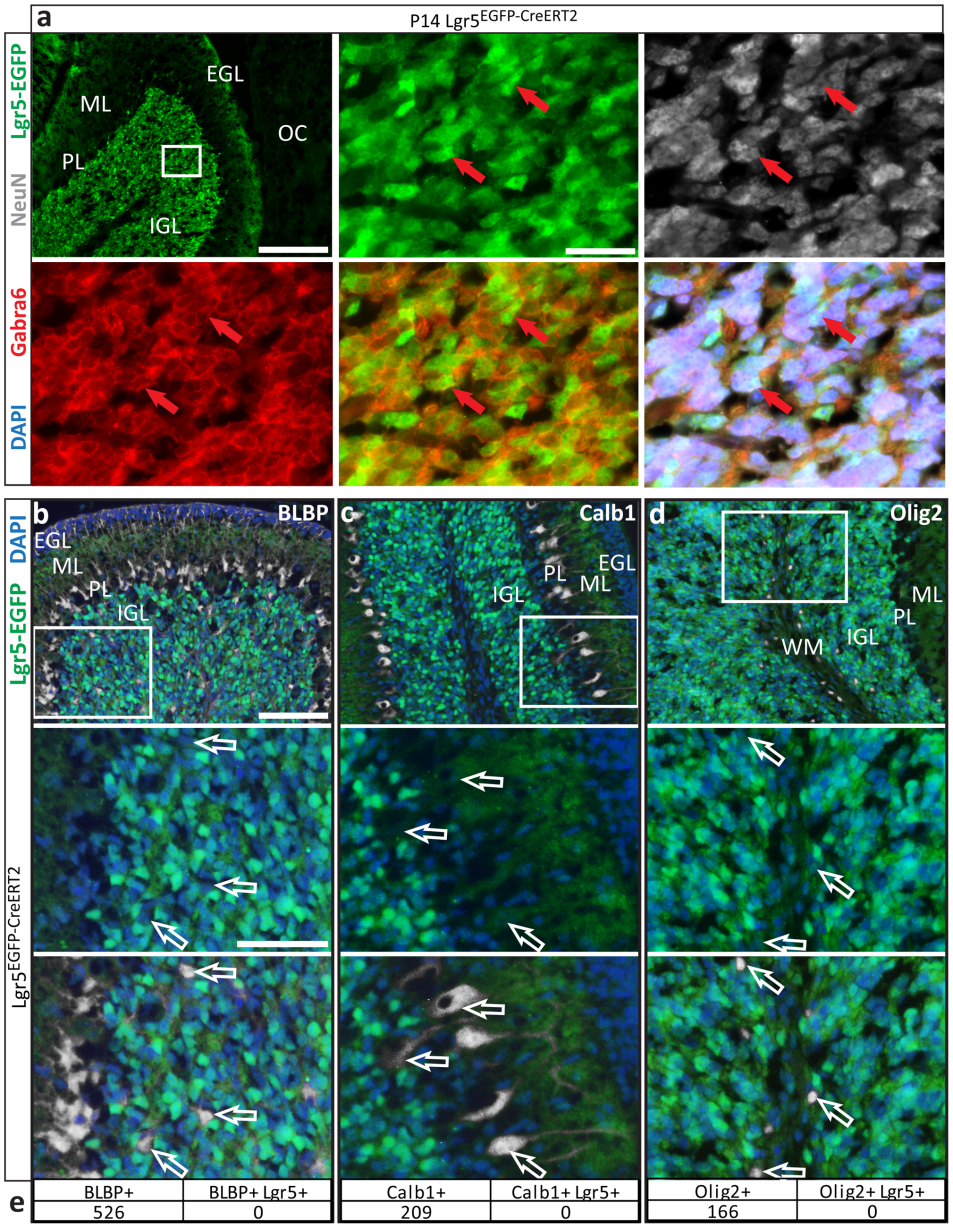
Postnatal Lgr5+ cells are exclusively cerebellar granule neurons. **a**) Sections from *Lgr5^EGFP-CreERT2^* P14 mouse brains were stained for EGFP to mark Lgr5+ cells, Gabra6 to identify cerebellar granule neuron, NeuN to identify neurons and DAPI to mark cell nuclei. Lgr5-EGFP+ cells are restricted to the internal granule layer (IGL) and all Lgr5+ cells are also positive for GABRA6 and NeuN. Lgr5-EGFP+ processes, presumably from Lgr5+ CGNs in the IGL can be seen in the molecular layer (ML). *Top left panel*, scale bar, 100 microns. Remaining 5 panels taken from marked area of top left panel. Scale bar, 25 microns. **b,c,d**) Representative images of *Lgr5^EGFP-CreERT2^* P10 and P14 mouse brains stained for Lgr5-EGFP and **b**) BLBP to identify Bergmann glia, **c**) Calb1 to identify Purkinje neurons and **d**) Olig2 to stain cells of the oligodendrocyte lineage. *Top panels*, scale bars, 100 microns. Bottom two panels taken from marked area of left panel. Scale bar, 50 microns. **e**) Cells that stained positive for one of the analyzed markers were counted and examined for Lgr5 expression. Numbers presented are aggregated from all sections analyzed (≥4 per marker). There was no overlap of Lgr5 with Calb1, BLBP or Olig2 in any sections analyzed. EGL –external granule layer; ML – molecular layer; PL – Purkinje layer; IGL – internal granule layer; OC – occipital cortex; WM – white matter tract.

To identify the specific cell population expressing Lgr5, we probed cerebellar sections from the *Lgr5^EGFP-CreERT2^* mouse with a panel of cerebellar cell lineage markers (Fig. 2). Lgr5 expressing cells were morphologically and geographically consistent with CGNs, nearly exclusively located in the internal granule layer in the cerebellum. Lgr5-positive cells were also positive for the neuronal marker, NeuN, and the mature granule neuron marker, gamma-aminobutyric acid type A (GABA(A)) receptor, alpha6 subunit (Gabra6) (Fig. 2a). Lgr5-positive cells were universally negative for the Bergmann glial marker, BLBP (Fig. 2b), the Purkinje neuron marker, Calbindin (Calb1) (Fig. 2c), and the oligodendrocyte lineage marker Olig2 (Fig. 2d) in all sections analyzed (Fig. 2e). Together, these findings indicate that Lgr5 is expressed in CGNs during postnatal development and that Lgr5 expression is exclusive to CGNs in the cerebellum during this period.

### Lgr5-positive cells in the postnatal cerebellum are lineage restricted

**Figure 3:**
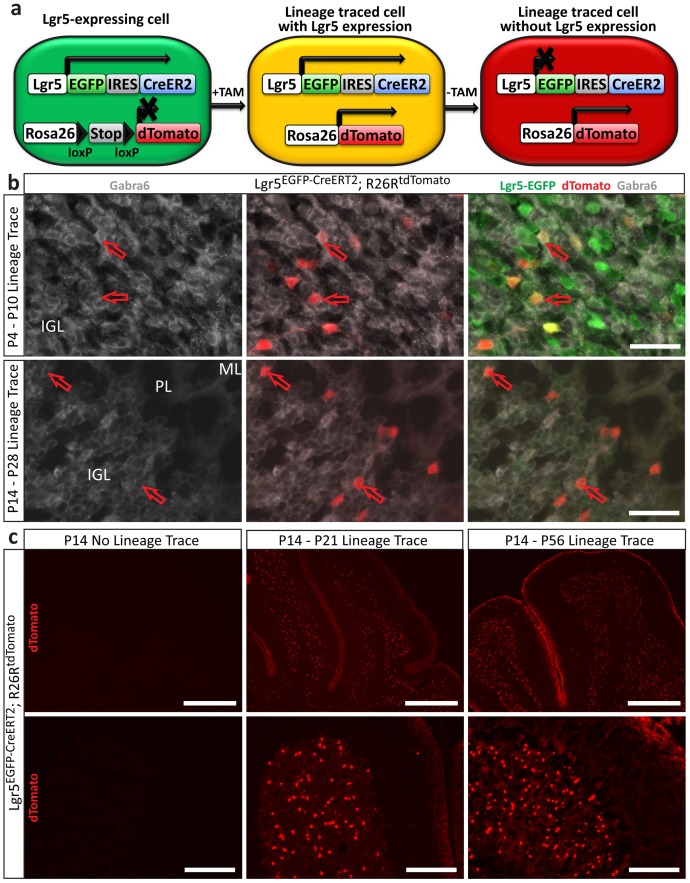
Lgr5-positive cells in the postnatal cerebellum are lineage-restricted. **a**) Schematic for lineage tracing design. Cells from *Lgr5^EGFP-CreERT2^; R26R^tdTomato^* mice express EGFP and CreER2 when the *Lgr5* locus is active. Addition of tamoxifen (TAM) activates CreER2, which removes the STOP element in the Rosa26-tdTomato locus permanently marking the cell and its progeny with tdTomato. Cells that maintain Lgr5 expression are double positive (yellow). **b**) Examples of lineage tracing time points. Cells that were once Lgr5+ (tdTomato+) were invariably Gabra6 positive, indicating granule neuron lineage restriction. *Top*: Lineage tracing was initiated at the beginning of Lgr5 expression in the IGL (P4) and cerebellums were analyzed at P10. *Bottom*: Lineage tracing was initiated near the end of Lgr5 expression in the IGL (P14) and analyzed at P28, when the *Lgr5* is no longer active in the IGL. Scale bar, 25 microns. **c**) Lineage tracing initiated at P14 was analyzed at extended time points, P21 and P56, demonstrating Lgr5+ cells at P14 survive and integrate into the adult IGL architecture. Scale bars, *top*: 400 microns, *bottom*: 100 microns.

**Figure 4:**
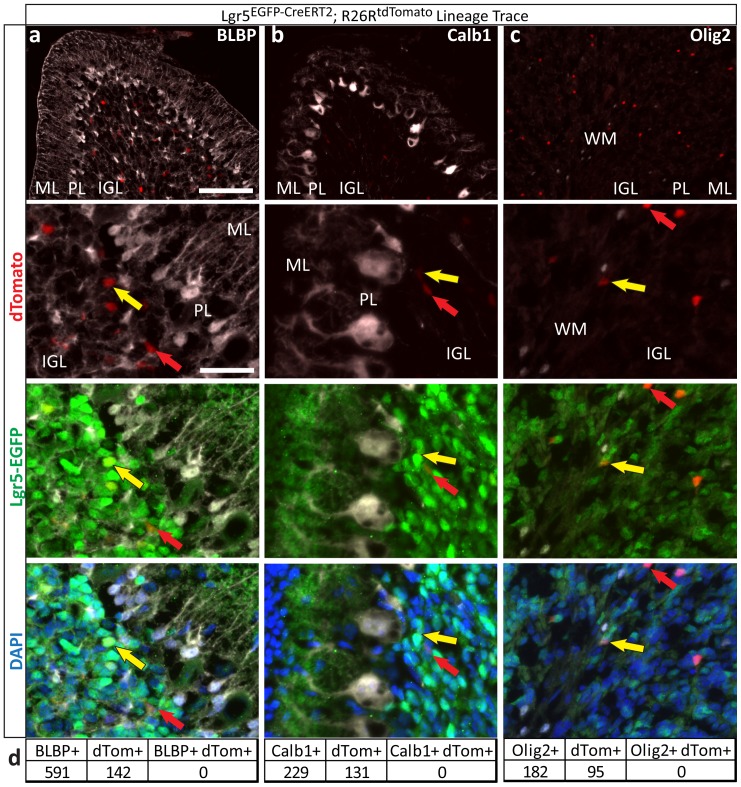
Cells previously Lgr5+ remain lineage restricted. In addition to tdTomato+ cells staining positive for Gabra6 (Fig. 3b), neither Lgr5+ cells nor cells previously Lgr5+ are positive for other lineage markers. Sections from *Lgr5^EGFP-CreERT2^; R26R^tdTomato^* mice that were lineage traced from P4 to P10 or P7 to P14 were stained for **a**) BLBP to identify Bergmann glia, **b**) Calb1 to identify Purkinje neurons and **c**) Olig2 to identify cell of the oligodendrocyte lineage. Representative images shown. Sections were also stained for Lgr5-EGFP and DAPI, while tdTomato marks lineage traced cells. **d**) Cells staining positive for a lineage marker or tdTomato were counted. Numbers shown are aggregated from all sections analyzed (≥4 per marker). There was no overlap of tdTomato and any lineage marker analyzed. Yellow arrows indicate examples of cells that are Lgr5+/tdTomato+ but lineage marker negative, while red arrows indicate examples of cells Lgr5-/tdTomato+ that are lineage marker negative. Scale bars, *top 3 panels*: 100 microns, *bottom 9 panels*: 25 microns. ML – molecular layer; PL – Purkinje layer; IGL – internal granule layer; WM – white matter tract.

In many tissues, Lgr5 marks multipotent stem cells; in contrast, we observed labeling of CGNs, which are terminally differentiated and lineage-restricted. To determine whether Lgr5-positive cells in the postnatal cerebellum included multipotent cells, we conducted lineage-tracing experiments by crossing *Lgr5^EGFP-CreERT2^* mice with *Rosa26-loxP-STOP-loxP-tdTomato* reporter mice (*Lgr5^EGFP-CreERT2^; R26R^tdTomato^*)[16]. The resulting animals express tamoxifen-inducible Cre recombinase (CreER^T2^) in Lgr5-positive cells, allowing permanent activation of a Cre-inducible tdTomato fluorescent protein in these cells and their progeny (Fig. 3a). The ability of Lgr5-positive cells to differentiate into other cells types can be determined by subsequent histological examination of the tdTomato-positive (lineage traced) cells. We initiated lineage tracing at P4, the first stage at which Lgr5 is detected in the internal granule layer, as well as at later time points (P7, P10, P14, P21 and P28). We analyzed cerebella from these mice 3, 7 and 14 days after lineage tracing initiation. Cells expressing Lgr5 at the time of tamoxifen administration generated only mature granule neurons at all time points (Figs. 3b and 4, and data not shown). Lineage tracing started at P21 or P28 resulted in no detectable tdTomato-positive cells at P28 or P35, respectively, indicating that the Lgr5 locus is no longer active in CGNs at these time points. Long-term lineage trace experiments revealed Lgr5-positive postnatal CGNs contributed to the adult cerebellar architecture, as expected, and continued to be lineage-restricted (Fig. 3c). Taken together, these results provide further evidence that Lgr5 specifically marks CGNs during a temporally restricted window and that these cells are long-lived and lineage-restricted.

### Lgr5-expressing CGNs are non-proliferative and post-mitotic

**Figure 5:**
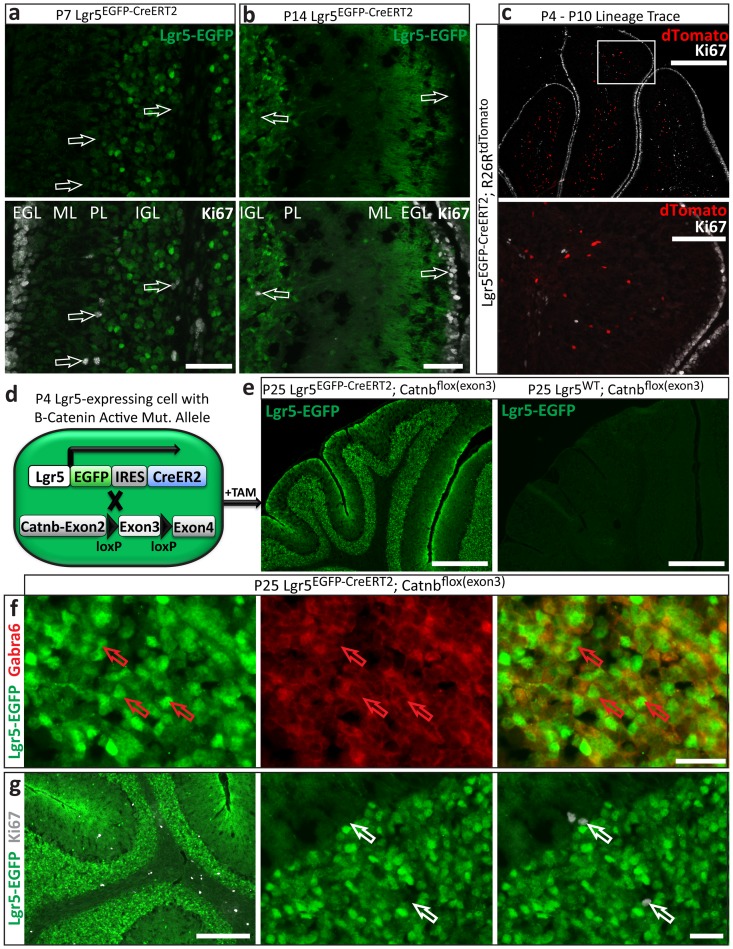
Lgr5 CGNs are non-proliferative and post-mitotic. **a, b**) Cerebellar sections from *Lgr5^EGFP-CreERT2^* mice were stained for the proliferation marker Ki67 and Lgr5-EGFP at both **a**) early (P7) and **b**) late (P14) stages of Lgr5 expression. Lgr5+ cells were uniformly negative for Ki67. Scale bars 50 microns. **c**) Lineage tracing was initiated at P4 in *Lgr5^EGFP-CreERT2^; R26R^tdTomato^* mice and cerebella analyzed for Ki67 at P10. Many other lineage trace time points were also analyzed (data not shown). Cells post-Lgr5 expression (tdTomato+) were uniformly negative for Ki67 in every time point analyzed. Note that immature granule neurons in the EGL in A-C were Ki67+, as expected. Scale bars for C *top*: 400 microns, *bottom*: 100 microns. **d**) *Lgr5^EGFP-CreERT2^* mice were crossed with *Catnb^flox(exon3)^* mice to yield *Lgr5^EGFP-CreERT2^*; *Catnb^flox(exon3)^* mice. Upon tamoxifen administration, Cre recombinase initiates expression of a constitutively active form of β-catenin in cells with an active *Lgr5* locus. **e**) Tamoxifen was administered at P4 and cerebella were analyzed at P25, when these mice die of other complications related to transgene expression. β-catenin over-activity led to prolonged Lgr5 expression in the IGL in *Lgr5^EGFP-CreERT2^*; *Catnb^flox(exon3^* mice (center panel), but not wild type (right panel). The normal architecture of the cerebellum was maintained in both conditions. Scale bars 400 microns. **f, g**) Lgr5+ CGNs from P25 *Lgr5^EGFP-CreERT2^*; *Catnb^flox(exon3)^* mice with tamoxifen administration at P4 were **f**) positive for Gabra6 and **g**) negative for Ki67. Scale bars, *bottom left*: 200 microns, *other 5 panels*: 25 microns.

To date, Lgr5 has been shown to mark cells that have the capacity for self-renewal and proliferation. However, mature CGNs are known to be post-mitotic. Therefore, we interrogated the proliferative potential of Lgr5-positive CGNs to understand if Lgr5 was expressed in a terminally differentiated and post-mitotic cell. Lgr5-positive cerebellar granule neurons were uniformly negative for Ki67, a marker of proliferation, at both early (Fig. 5a) and late stages of Lgr5 expression (Fig. 5b). Cells that were previously Lgr5-positive CGNs, as identified by lineage tracing, were also negative for Ki67 (Fig. 5c). However, immature granule neurons in the EGL, which are not Lgr5 positive, are highly proliferative and accordingly stained intensely for Ki67 (Fig. 5a-c).

To further investigate if Lgr5-postive CGNs had the capacity for self-renewal or proliferation, we interrogated if over-activation of Wnt signaling could stimulate proliferation of Lgr5-positive CGNs. In other tissue types, constitutively active Wnt signaling increases proliferation of Lgr5-positive populations [21], [22] and can cause tumor formation [23]. We crossed *Lgr5^EGFP-CreERT2^* mice with *Catnb^flox(exon3)^* mice [17] to yield *Lgr5^EGFP-CreERT2^*; *Catnb^flox(exon3)^* mice, in which Cre recombinase initiates expression of a constitutively active form of β-catenin in Lgr5-positive cells following treatment with tamoxifen (Fig. 5d). β-catenin is the key downstream node for canonical Wnt signaling and increasing its activity increases Wnt signaling-dependent cellular responses through transcriptional regulation. We found that tamoxifen administration at P4 led to a robust and prolonged expression of Lgr5 in CGNs (Fig. 5e, f). However, this increase in Lgr5 and Wnt signaling did not disrupt normal architecture of the cerebellum (Fig. 5e), did not disrupt Lgr5-positive CGN lineage-restriction (Fig. 5f) or initiate proliferation in these cells as has been shown in other systems [22] (Fig. 5g). These results further support the notion that Lgr5 does not regulate proliferation or self-renewal in CGNs.

### Maturing CGNs progress through a transient Lgr5-positive phase

**Figure 6:**
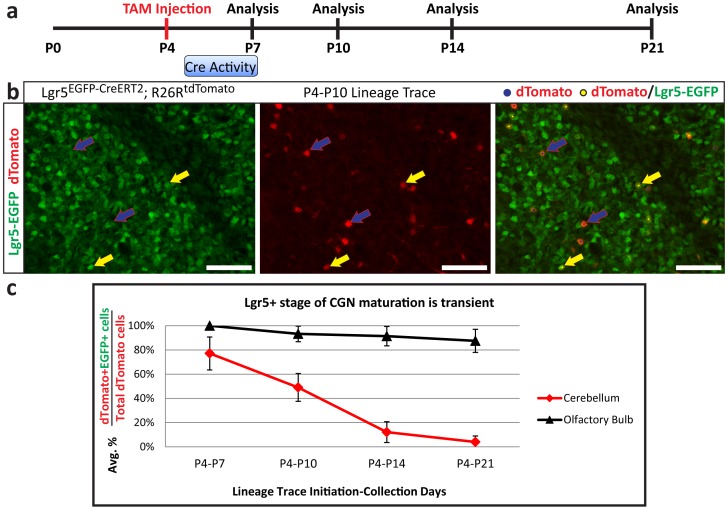
Maturing CGNs progress through a transient Lgr5 phase resulting in mature CGNs. **a**) Schematic of lineage tracing time course. Tamoxifen (TAM) was injected at P4 into several large litters and brains from pairs of wildtype (WT) and *Lgr5^EGFP-CreERT2^; R26R^tdTomato^* mice from each litter, were analyzed at the indicated time points. Period of predicted Cre activity is indicated. **b**) Multiple sections from each brain analyzed were stained for Lgr5-EGFP to mark cells currently Lgr5+, while tdTomato expression marked previously Lgr5+ cells that had undergone recombination. Shown is an example from a P10 brain that had lineage tracing initiated at P4. Yellow dots indicate cells marked as Lgr5+/tdTomato+ by image analysis algorithm (yellow arrows indicate examples), while blue dots indicate cells marked as Lgr5-/tdTomato+ (blue arrow with red outline point out examples). Scale bars, 50 microns. **c**) Quantification from an image analysis algorithm of all sections analyzed.

CGNs are a relatively homogenous population of neurons that progress through defined stages of development regulated by distinct phases of temporally regulated gene expression [6], [24]. Lgr5 is expressed in CGNs once they enter the IGL, and at a population level Lgr5 is expressed in CGNs in the IGL from P4 to P21. However, as individual CGNs progress through maturation at different times during the postnatal time period based on when they exit the proliferative stage in the EGL, we sought to determine the duration of Lgr5 expression in individual CGNs during maturation *in vivo.* We again utilized *Lgr5^EGFP-CreERT2^; R26R^tdTomato^* mice to conduct a lineage tracing time course initiated at the first signs of Lgr5 expression in CGNs at P4 and analyzed cerebella for tdTomato-positive cells at multiple time points until P21, when the Lgr5 locus is no longer active (Fig. 6a). We calculated the ratio of tdTomato-positive cells that were also positive for Lgr5 to those that were solely tdTomato-positive in order to understand how quickly Lgr5-expression was turned off in cells that were Lgr5-positive at the time of lineage trace initiation. When initiating lineage tracing at P4, we found that few cells had gone through both recombination and production of the tdTomato protein by P7, as anticipated. Once Lgr5-positive cells are exposed to tamoxifen, it can take up to 24 hours before recombination and transcription occurs, and tamoxifen can remain in the system, maintaining Cre recombinase activity for a further 48 hours [25]. Therefore, we expected Lgr5-positive cells to be labeled from P4-P7 (Fig 6a), and, with considerably more cells expressing Lgr5 at P7, we anticipated most lineage-traced cells would be derived from the later days of this time period. Of the few cells that were tdTomato-positive at P7, nearly 20 percent were already Lgr5-negative. By P10, 50 percent of tdTomato-positive cells were Lgr5-negative (Fig. 6b,c) and this increased to nearly 90 percent negative at P14. These results suggest that the phase of Lgr5 expression in individual CGNs during maturation is transient (Fig 6c). Based on the length of Cre activity and the sharp drop in tdTomato-positive/Lgr5-positive cells, we predict that Lgr5 is expressed during a period of 3-5 days in an individual CGN. Given that the half-life of the EGFP reporter is greater than 24 hrs [26], the length of time that Lgr5 is active in a given cell is likely even shorter than 3-5 days predicted by the EGFP reporter. This is consistent with the observation that Lgr5 acts in a short period to provide a temporary elevation in Wnt signaling activity [15]. The short period of Lgr5 expression in individual cells and the high percentage of CGNs that are Lgr5-positive at any given point during the postnatal period supports the idea that Lgr5 expression occurs in most, if not all, CGNs during their maturation.

### Expression of the Lgr5 ligand, R-spondin 1, coincides with Lgr5 expression in CGN maturation

**Figure 7:**
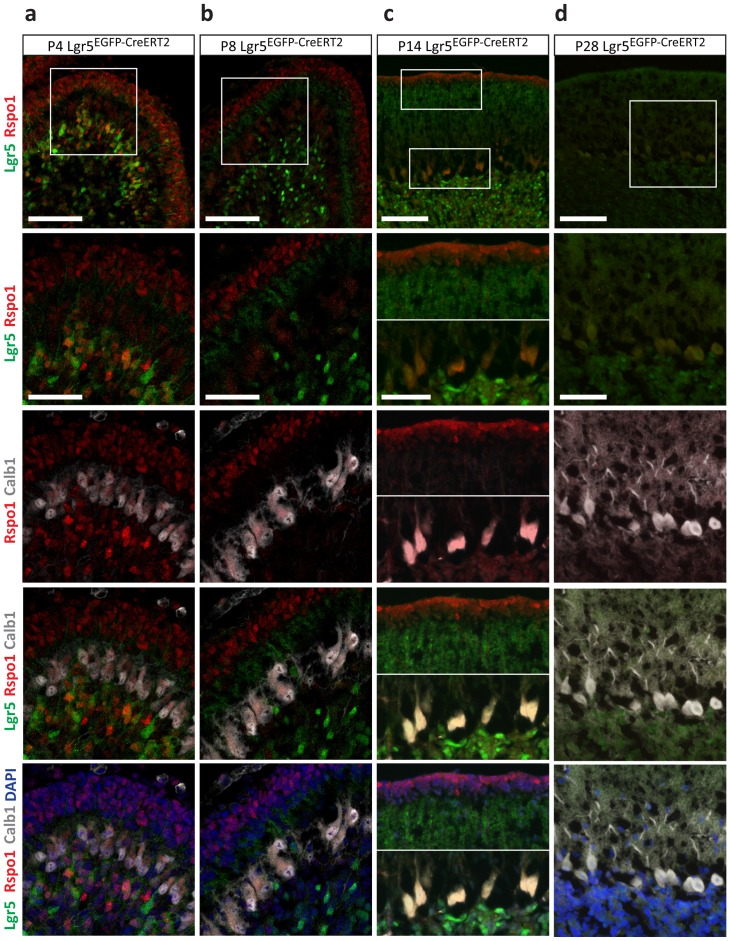
The source of the Lgr5 ligand, R-Spondin1, temporally varies with CGN maturation. Cerebellar sections from *Lgr5^EGFP-CreERT2^* mice at increasing developmental time points **a**) P4, **b**) P8, **c**) P14 and **d**) P28 were stained for Lgr5-EGFP, Rspo1, Calb1 and DAPI. Top row panels are representative images while boxes indicate magnified regions in bottom 4 rows. Scale bars, *top row*: 100 microns, *bottom 4 rows*: 50 microns.

**Figure 8:**
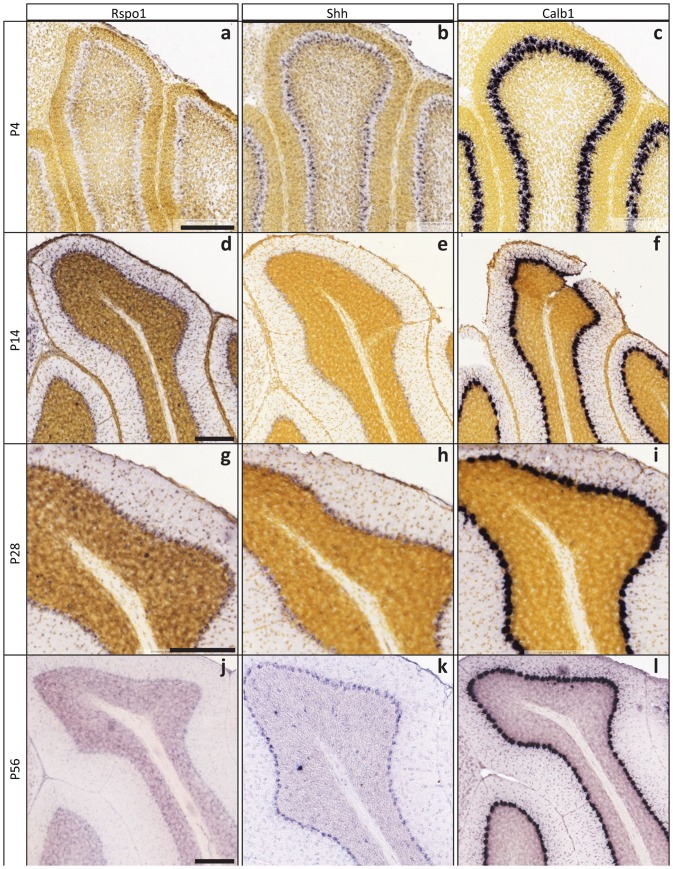
Allen Developing Mouse Brain Atlas ISH data supports Rspo1 data. RNA in situ hybridization data from the Allen Developing Brain Atlas was queried for Rspo1 (**a, d, g, j**)(http://developingmouse.brain-map.org/gene/show/82848), Shh (**b, e, h, k**) (http://developingmouse.brain-map.org/gene/show/20186) and Calb1 (**c, f, i, l**) (http://developingmouse.brain-map.org/gene/show/12092) at P4, P14, P28 and P56. Sagitttal sections from indicated time points were used. Representative images of each gene at each time point are presented. Calb1 serves as a consistent positive control marker for Purkinje neurons in the Purkinje layer, while Rspo1 and Shh RNA transcription varies over time within this layer. Feulgen-HP yellow DNA stain was used as a counter stain for all time points except P56 to provide tissue context to the ISH signal (violet). Scale bars, 200 microns.

In epithelial tissues, activation of the Wnt pathway through the Lgr5 receptor is initiated by the R-Spondin family of ligands [14]. Therefore, we stained for Rspo1 at various stages of CGN development to understand if this ligand was potentially regulating Lgr5 activation in CGNs. We found Rspo1 expression mirrored the onset and loss of Lgr5 expression (Figs. 7 and 8). The initial cellular source of Rspo1 appeared to be CGNPs in the EGL, with expression present by P4 (Fig. 7a). As the EGL shrunk due to CGNP differentiation and migration into the IGL, Purkinje neurons, which are known to secrete Shh during CGN development [5], began to express Rspo1, exhibiting maximal staining between P8 and P14 (Fig. 7b–c). Rspo1 expression was barely detectable at P28, its expression loss coinciding with the loss of Lgr5 expression (Fig. 7d).

Purkinje neuron secretion of the Lgr5 ligand, Rspo1, and potentially a transition of Shh to Rspo1 secretion at these time points was supported by the Allen Developing Mouse Brain Atlas (RRID:nif-0000–00509) [27]. The Allen Developing Mouse Brain Atlas contains a database of RNA *in situ* hybridization (ISH) for nearly 2000 genes across seven stages of brain development. Using this database, we found that Rspo1 expression was low at P4 and much higher at P14, supporting our immunofluorescence data (Fig. 8a, d). In contrast, Shh was expressed in Purkinje neurons at high levels at P4, but this expression was lower at P14 (Fig. 8b, e). Purkinje neurons regained expression of Shh at P28 and continued expressing Shh through adulthood (P56) (Fig. 8h, k), while Rspo1 expression was completely abolished by P56 (Fig. 8j). Purkinje neurons secrete Shh, which acts to stimulate proliferation of CGN precursors in the EGL [5], but our results demonstrate that Purkinje neurons may also have a role in the next phase of CGN differentiation by activating the Wnt pathway through Rspo1 expression. Indeed, migrating CGNs must past directly through the layer of Purkinje neurons on their journey to the IGL, exposing them directly to this secreted ligand.

## Discussion

Nearly 15 years ago, two reports using a panel of homogenized tissue RNA showed the then-orphan receptor Lgr5 (also termed FEX, HG38, GPR49, and GPR67) had detectable expression in the brain [28], [29]. However no detailed characterization of Lgr5 expression in the developing or adult brain has since been published. What initially started as a search for Lgr5 expression in neural stem and progenitor cells led us to the surprising discovery that Lgr5 is expressed in maturing CGNs, the most prevalent neuronal population in the mammalian brain. This is surprising because Lgr5 is a well-characterized marker of epithelial stem and progenitor cells in somatic tissues, but mature CGNs are both non-epithelial and post-mitotic. However, CGNs progress through a well-organized differentiation process during postnatal development and are dependent on Wnt signaling for full maturation, indicating a potential purpose for this Lgr5 expression [3], [6].

In this report, we demonstrate that Lgr5 is expressed in CGNs in the IGL during postnatal cerebellar development, and that Lgr5-positive CGNs are lineage-restricted and post-mitotic, even in the presence of forced activation of the Wnt/β-catenin pathway. We find that Lgr5 is expressed from P4-P21 with no expression in the adult cerebellum. CGNs migrate into the IGL and mature continuously during the same period of Lgr5 expression, suggesting a role for Lgr5 in the maturation of CGNs. Based on the very high percentage of CGNs that are Lgr5-positive from P7 to P14 and the phasic nature of Lgr5 expression indicated by our results, we conclude it is likely that nearly all CGNs go through a short period of Lgr5 expression, which we speculate may initiate or enhance Wnt signaling during the Wnt dependent phase of synaptogenesis and maturation.

In further support of this possibility, Rspo1, the Lgr5 ligand that triggers downstream Wnt signaling is also expressed in the postnatal cerebellum transiently during CGN maturation. Interestingly, the cell types that express Rspo1 during this window vary with the changing architecture of the cerebellum during postnatal development. Early in development, when most granule neurons are immature precursors and the EGL and IGL are in close proximity, CGNPs express Rspo1. However, as CGNPs mature, the EGL diminishes and is separated from the IGL and the maturing CGNs by an expanding molecular layer. At this point, Purkinje neurons that are directly adjacent to the IGL begin to express Rspo1, suggesting that the Rspo1/Lgr5 program that controls Wnt signaling in other tissue types is also tightly regulated in the developing cerebellum.

During maturation, CGNs respond to Wnt signaling to form synapses with mossy fiber axons and Purkinje neurons [8], [9]. This response is mediated through the binding of Wnt-7a to the Frizzled-5 (Fz5) receptor [10]. Lgr5 has been shown in other tissues to be a Wnt pathway co-receptor that recruits and binds the LRP-Frizzled receptor complex, and subsequently responds to Wnt ligands to increase signaling in the Wnt/β-catenin pathway [14]. Therefore, Lgr5 may be acting as a co-receptor within this Wnt-7a responsive complex to facilitate CGN maturation. It is possible, however that Lgr5 may have a completely novel role in CGNs outside of its known roles in epithelial stem and progenitor cells.

These results raise the possibility that Lgr5 may not only function in stem and progenitor cells, but may have a more general role as a regulator of cell fate. In fact, a recent report from our group demonstrates that Lgr5 is also constitutively expressed in a subpopulation of mature retinal amacrine cells, interneurons in the mammalian eye, providing additional evidence of a role outside of epithelial stem and progenitor cells [30]. *Lgr5^EGFP-CreERT2^* homozygous mice die within 24 hours of birth, which currently limits our ability to study the effect of Lgr5 knockout on CGNs *in vivo.* Future studies using conditional Lgr5 knockout mice could be conducted to further study the role of Lgr5 in nervous system tissue.

Wnt signaling is critical in many developing and adult tissues, and disruption or over-activation of the Wnt pathway can lead to severe abnormalities in many tissues, including the cerebellum [11], [13]. Disruption of Lgr5 in the intestine led to rapid and complete loss of intestinal crypts comparable to loss of Wnt signaling [14], while overexpression of Rspo1 led to rapid expansion of intestinal crypts, demonstrating how important these Wnt signaling regulators are to the tightly regulated Wnt pathway. Our study provides evidence that Lgr5 and Rspo1 have roles outside of epithelial stem and progenitor cells, and provides novel insight into the function and regulation of Wnt signaling in cerebellar development.

## Supporting Information

S1 Figure **Lgr5 is not expressed in neurogenic regions.** Sections from the ventricular zone of E18.5 *Lgr5^EGFP-CreERT2^* mice were co-stained for EGFP to mark Lgr5+ cells, and Pax6, Tbr2 and Nestin to mark neurogenic cell types (top 3 rows). Sections from the sub-ventricular zone and dentate gyrus of P7 *Lgr5^EGFP-CreERT2^* mice were co-stained for EGFP to mark Lgr5+ cells, and prominin-1 and Nestin, respectively, to mark neurogenic cells. There was no overlap of Lgr5 with Pax6, Tbr2, Nestin or prominin-1 in any sections analyzed. Scale bars, 100 microns.
(TIF)
