# Supplementary figures and images for: Lgr5 Marks Post-Mitotic, Lineage Restricted Cerebellar Granule Neurons during Postnatal Development
Source: PLoS One. 2014 Dec 10;9(12):e114433. doi: 10.1371/journal.pone.0114433 (PMC4262395; doi:10.1371/journal.pone.0114433)

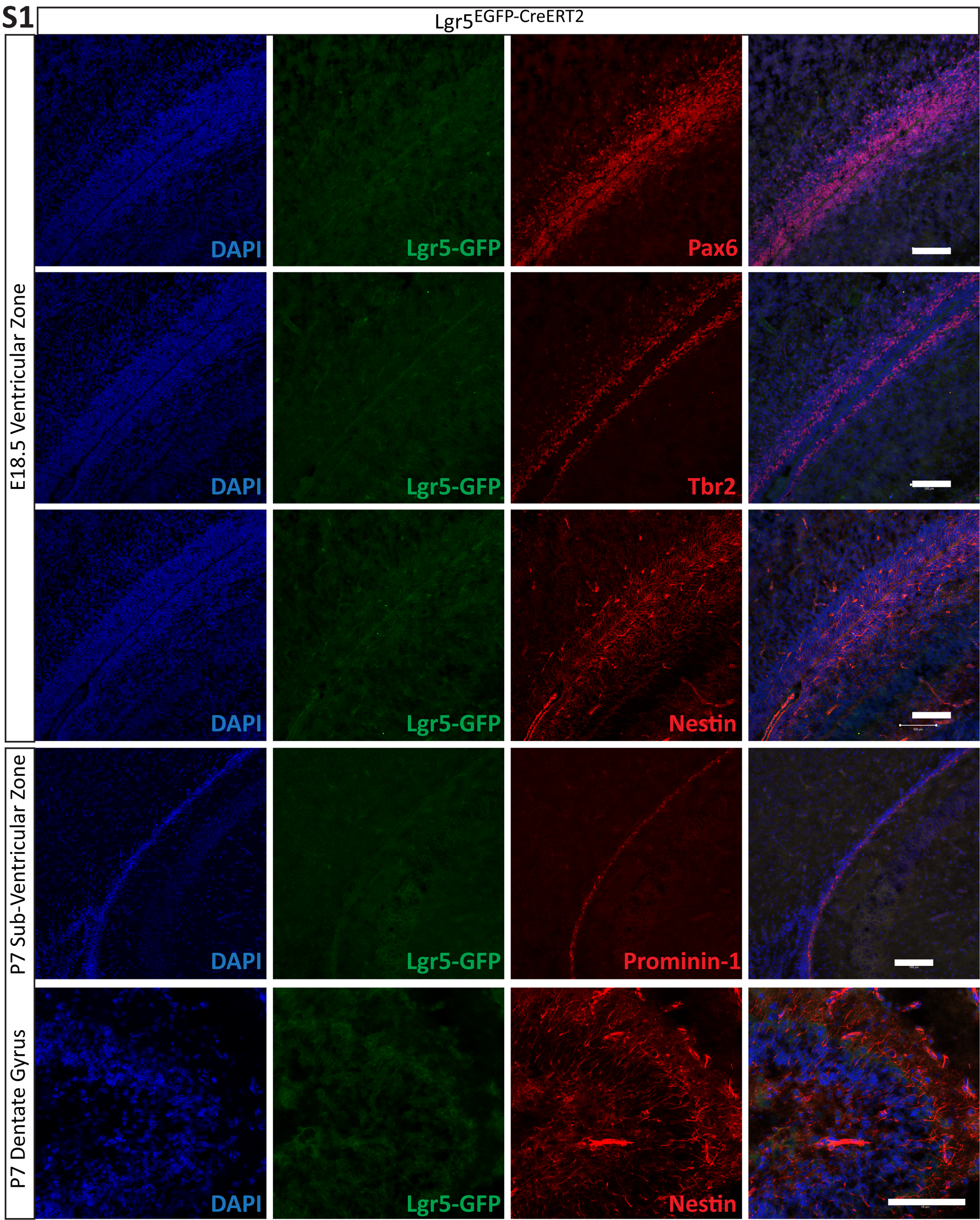

Supplement: S1 Figure — Lgr5 is not expressed in neurogenic regions. Sections from the ventricular zone of E18.5 Lgr5EGFP-CreERT2 mice were co-stained for EGFP to mark Lgr5+ cells, and Pax6, Tbr2 and Nestin to mark neurogenic cell types (top 3 rows). Sections from the sub-ventricular zone and dentate gyrus of P7 Lgr5EGFP-CreERT2 mice were co-stained for EGFP to mark Lgr5+ cells, and prominin-1 and Nestin, respectively, to mark neurogenic cells. There was no overlap of Lgr5 with Pax6, Tbr2, Nestin or prominin-1 in any sections analyzed. Scale bars, 100 microns. (TIF) [file pone.0114433.s001.tif]
